# Molecular analysis of blood-associated pathogens in European wildcats (*Felis silvestris silvestris*) from Germany

**DOI:** 10.1016/j.ijppaw.2022.08.012

**Published:** 2022-09-03

**Authors:** Maria Sophia Unterköfler, Josef Harl, Bita Shahi Barogh, Joachim Spergser, Kristýna Hrazdilová, Franz Müller, Diana Jeschke, Ole Anders, Peter Steinbach, Hermann Ansorge, Hans-Peter Fuehrer, Mike Heddergott

**Affiliations:** a Institute of Parasitology, Department of Pathobiology, University of Veterinary Medicine Vienna, Austria; b Institute of Pathology, Department of Pathobiology, University of Veterinary Medicine Vienna, Austria; c Institute of Microbiology, Department of Pathobiology, University of Veterinary Medicine Vienna, Austria; d Department of Chemistry and Biochemistry, Mendel University, Brno, Czech Republic; e Faculty of Medicine in Pilsen, Biomedical Center, Charles University, Pilsen, Czech Republic; f Working Group for Wildlife Research, Justus-Liebig-University Giessen, Germany; g Senckenberg Museum of Natural History Görlitz, Germany; h Nationalpark Harz, Sankt Andreasberg, Germany; i Faculty of Chemistry, University of Göttingen, Germany; j Musée National d’Histoire Naturelle, Luxembourg; k International Institute Zittau, Technical University Dresden, Zittau, Germany

**Keywords:** Vector-borne disease, *Mycoplasma*, *Hepatozoon*, *Cytauxzoon*, *Bartonella*

## Abstract

European wildcats (*Felis silvestris silvestris*) have not been investigated in large numbers for blood-associated pathogens in Germany, because wildcats, being a protected species, may not be hunted, and the collection of samples is therefore difficult. Thus, spleen tissue and whole blood from 96 wildcats from Germany found as roadkill or dead from other causes in the years 1998–2020 were examined for the prevalence of blood associated pathogens using molecular genetic tools. PCR was used to screen for haemotrophic *Mycoplasma* spp., *Hepatozoon* spp., *Cytauxzoon* spp., *Bartonella* spp., Filarioidea, Anaplasmataceae, and Rickettsiales, and positive samples were subsequently sequenced. Phylogenetic analyses were performed for *Mycoplasma* spp. and *Hepatozoon* spp. by calculating phylogenetic trees and DNA haplotype networks. The following pathogens were found: *Candidatus* Mycoplasma haematominutum (7/96), *Mycoplasma ovis* (1/96), *Hepatozoon silvestris* (34/96), *Hepatozoon felis* (6/96), *Cytauxzoon europaeus* (45/96), and *Bartonella* spp. (3/96). This study elucidates the prevalence of blood-associated pathogens in wildcats from Germany.

## Highlights

• European wildcats from Germany carry different blood-associated pathogens.
• Pathogens can also affect domestic cats.
• Transmision by vectors or other transmisison routes are possible.

## Graphical abstract

**Figure:**
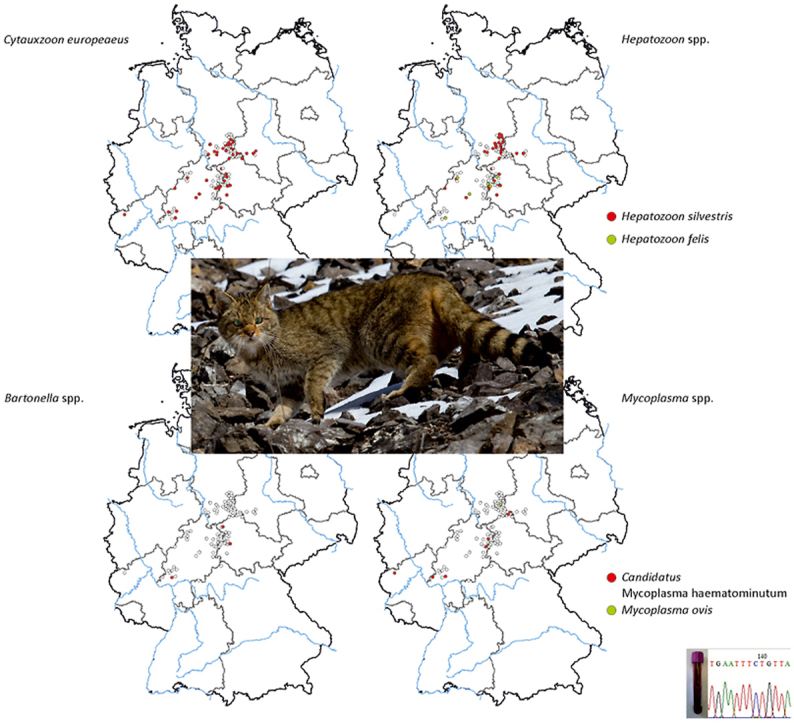

## 1 Introduction

Arthropod vectors are responsible for the transmission of several blood-associated parasites and bacteria, which can cause disease in wild and domestic animals, as well as in humans. Their relevance is increasing due to climate change driven migration into regions that are more temperate. Other factors promoting the introduction and spread of vector-borne pathogens (VBP) include, for instance, globalization, habitat change, loss of biodiversity, and pollution (Harrus and Baneth, 2005; Aguirre, 2009). Different wildlife species have different effects on the density of vectors (Takumi et al., 2019), and the role of wildlife in the transmission of VBP to humans and pets is not yet fully elucidated (Mackenstedt et al., 2015). Wild carnivores are important for the maintenance of the sylvatic cycle, therefore understanding the epidemiology of their VBP is crucial (Battisti et al., 2020). Wild canids and felids can spread disease-causing pathogens to their domestic counterparts and vice versa. The close relationship of pet dogs and cats to humans increases the risk of the emergence of zoonotic disease (Otranto et al., 2015).

The European wildcat (*Felis silvestris* *silvestris*) is closely related to the domestic cat (*Felis silvestris catus*) and inhabits pristine forests in different parts of Europe, with one main population group in Germany (Mattucci et al., 2016). Since the 17th their population has been significantly reduced as a result of human activities century, and the species is now listed as endangered (Heddergott et al., 2018; Meinig et al., 2020). However, species protection and habitat restoration has enabled the wildcat to thrive again, and there is now an estimated population size of 5000–10000 individuals (Balzer et al., 2018; European Topic Centre on Biological Diversity, 2019). Nonetheless, anthropogenic habitat fragmentation and possible genetic introgression from domestic cats still threaten their genetic integrity, and conservation measures are still essential (Hertwig et al., 2009; Eckert et al., 2010; Witzenberger and Hochkirch, 2014; Mattucci et al., 2016). Understanding the prevalence of potential pathogens in wildcat populations can be important in this context (Poirson and Dutilleul, 2014).

Only few studies on blood-associated parasites and bacteria in these animals are available due to their secretive lifestyle and, compared to the ubiquitous red fox (V*ulpes vulpes*), small population size, making it difficult to obtain samples from these animals (Hodžić et al., 2018a). Apicomplexan parasites such as *Hepatozoon* spp., *Cytauxzoon* spp., and *Babesia* spp. are frequently found in wildcats (Zaeemi et al., 2015; Gallusová et al., 2016; Veronesi et al., 2016; Hodžić et al., 2018a; Hornok et al., 2022). *Hepatozoon* spp. use a wide range of vertebrates as intermediate hosts and hematophagous invertebrates as definitive hosts and vectors. The transmission to their vertebrate host is mainly through ingestion of the vector but can also happen through predation via tissue cysts or transplacental transmission (Nordgren and Craig, 1984; Johnson et al., 2009; Baneth et al., 2013). Among the Hepatozoidae, *Hepatozoon felis* is the predominant species found in cats and wildcats, but *H. silvestris* and have also been described (Giannelli et al., 2017a; Hodžić et al., 2017). The vectors of feline hepatozoonosis are unknown (Hodžić et al., 2017). Among the family Theileriidae, *Cytauxzoon felis*, *C. manul,* and three newly described species, namely *C. europaeus*, *C. otrantorum,* and *C. banethi*, have been reported from felids. The tick species *Amblyomma americanum* and *Dermacentor variabilis* were identified as vectors for *C. felis* in America (Blouin et al., 1984; Reichard et al., 2010). The vector is not known for European *Cytauxzoon* spp., but *Ixodes ricinus* has been suggested due to its high abundance (Panait et al., 2021b). Bacterial pathogens transmitted by vectors to domestic cats include *Bartonella* spp., *Anaplasma* spp., *Rickettsia* spp., and *Mycoplasma* spp. (Lappin, 2018). Of these, *Mycoplasma* spp. has also been reported in wildcats (Willi et al., 2007; Hodžić et al., 2018a). *Candidatus* Mycoplasma haemominutum was first described by Foley and Pedersen (2001); it affects domestic cats as well as wild felids (Foley and Pedersen, 2001; Willi et al., 2007; Cerreta et al., 2022). Oren (2017) suggested correcting the name to *Candidatus* Mycoplasma haematominutum for the sake of linguistic accuracy.

Reports of Filarioidea in wildcats are only sporadic and the role of wildcats in the maintenance of the sylvatic cycle of Filarioidea, such as *Dirofilaria immitis,* is considered to be low (Penezić et al., 2014; Ionică et al., 2017).

In the present study, we screened blood and spleen tissue of wildcats from Germany for blood-associated pathogens using molecular genetic methods, to estimate the possibility of pathogen transmission between wildcats and domestic cats by vectors or other transmission routes.

## 2 Materials and methods

### 2.1 Sample collection

**Fig. 1:**
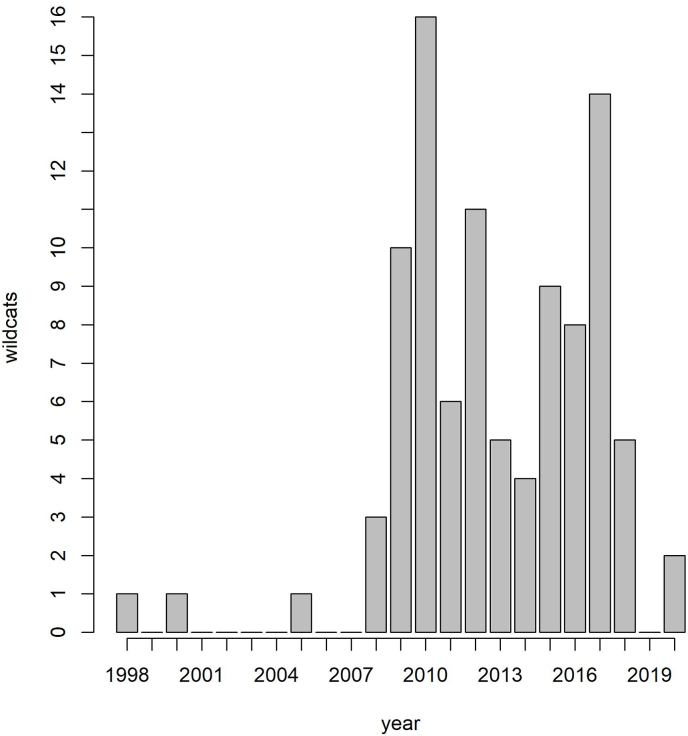
Distribution of wildcat samples in total number of wildcats (y-axis) collected per year (x-axis).

**Fig. 2:**
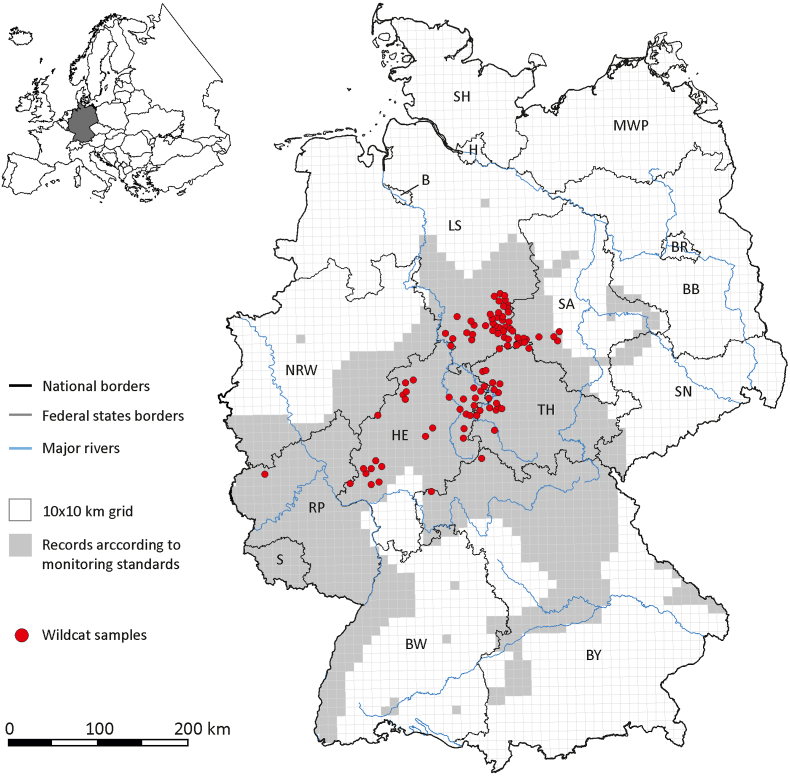
Geographic origin of the 96 European wildcats (*Felis silvestris*) from Germany included in this study. The gray area represents the geographic distribution of wildcats in Germany according to the National FFH Report 2019, plotted on the 10 × 10 km reference grid ETRS89-LAEA5210 EEA according to a compilation of the German Federal Agency for Nature Conservation (BfN) and monitoring data of the federal states (Bundesamt für Naturschutz, 2020). Abbreviations: Brandenburg (BB), Bremen (B), Berlin (BR), Baden-Württemberg (BW), Bavaria (BY), Hamburg (H), Hesse (HE), Mecklenburg-West Pomerania (MWP), Lower Saxony (LS), North Rhine-Westphalia (NRW), Rhineland-Palatinate (RP), Schleswig-Holstein (SH), Saarland (S), Saxony (SN), Saxony-Anhalt (SA) and Thuringia (TH).

Between 1998 and 2020, 96 wildcats found as roadkill or dead from other causes were collected in Germany (Fig. 1) and stored at −20 °C until necropsy. The individuals originated from six federal states: Bavaria (n = 2), Hesse (n = 30), Lower Saxony (n = 37), Rhineland-Palatinate (n = 1), Saxony-Anhalt (n = 4), and Thuringia (n = 22) (Fig. 2). During dissection, 1–5 ml of blood were collected from 55 individuals and spleen samples from 41 individuals. The samples were stored at −20 °C until final processing at the University of Veterinary Medicine, Vienna. The cats were classified as wildcats according to the intestinal index (Braunschweig, 1963) and cranial index (Schauenberg, 1969). Individuals, where classification was not clear or not possible due to severe destruction, were genetically tested (Steyer et al., 2016). The age determination of the wildcats was based on the growth lines in the enamel of a mandibular canine (Ansorge, 1995; Heddergott et al., 2016). According to Piechocki and Stiefel (1988), the cats were assigned to two age classes: juvenile/subadult (≤24 months; none or one growth line) and adult (≥25 months; two or more growth lines).

### 2.2 DNA extraction, PCR amplification, and sequencing

DNA was isolated from spleen samples and whole blood using the QIAGEN DNeasy Blood and Tissue kit (QIAGEN, Hilden, Germany). Samples were incubated at 56 °C overnight and processed according to the manufacturer's protocol. Samples were screened for the presence of various blood-associated pathogens using specific broad-range PCR assays (Table 1) targeting the following fragments: *Mycoplasma* spp. Within the 16 S rRNA gene, and if positive, a larger fragment of the 16 S rRNA gene; Piroplasmida and other Apicomplexa within the 18 S rRNA gene, and if positive, for *Hepatozoon* spp. or *Cytauxzoon* spp. a larger fragment of the 18 S rRNA gene; and for *Cytauxzoon* spp. additionally the cytochrome *b* gene (*CytB*); *Bartonella* spp. Within the 16 S–23 S rRNA gene, and if positive, the citrate synthase gene (*gltA*); *Rickettsia* spp. the 23 S–5S rRNA gene; Anaplasmataceae within the 16 S rRNA gene; and Filarioidea targeting a fragment of the mitochondrial cytochrome *c* oxidase subunit I gene (*COI*). Positive and negative controls were used to validate results. PCR products were analyzed by electrophoresis in 2% agarose gels stained with Midori Green Advance DNA stain (Nippon Genetics Europe, Germany). Positive samples were sent to a commercial company (LGC Genomics GmbH, Germany) for sequencing using amplification primers.

**Table 1:** Oligonucleotide sequences of primers used in the present study.

Target organism (genetic marker)	Primer sequences (5′→3′)		Product size	Reference
*Mycoplasma* spp. (16 S rRNA)	HBT-F: ATA CGG CCC ATA TTC CTA CG		600 bp	Criado-Fornelio et al. (2003)
	HBT-R: TGC TCC ACC ACT TGT TCA			
*Mycoplasma* spp. (16 S rRNA)	UNI_16 S_mycF: GGC CCA TAT TCC TAC GGG AAG CAG CAG T		1000 bp	Volokhov et al. (2011)
	UNI_16 S_mycR: TAG TTT GAC GGG CGG TGT ACA AGA CCT G			
*Hepatozoon* spp. (18 S rRNA)	H14Hepa18SFw: GAA ATA ACA ATA CAA GGC AGT TAA AAT GCT		620 bp	Hodžić et al. (2015)
	H14Hepa18SRv: GTG CTG AAG GAG TCG TTT ATA AAG A			
Piroplasmida (18 S rRNA)	BTH-1F: CCT GAG AAA CGG CTA CCA CAT CT		700 bp	Zintl et al. (2011)
	BTH-1R: TTG CGA CCA TAC TCC CCC CA			
	GF2: GTC TTG TAA TTG GAA TGA TGG		561 bp	
	GR2: CCA AAG ACT TTG ATT TCT CTC			
*Cytauxzoon* spp. (18 S rRNA)	7549 F: GTC AGG ATC CTG GGT TGA TCC TGC CAG		1726 bp	Millán et al. (2007)
	7548 R: GAC TGA ATT CGA CTT CTC CTT CCT TTA AG			
	Cyt-SSU-F2: CAT GGA TAA CCG TGC TAA TTG		1335 bp	Panait et al. (2021b)
	Cyt-SSU-R4: AGG ATG AAC TCG ATG AAT GCA			
*Cytauxzoon* spp. (C*ytB*)	Cytaux_cytb_F1: CTT AAC CCA ACT CAC GTA CC		1434 bp	Schreeg et al. (2013)
	Cytaux_cytb_R3: GGT TAA TCT TTC CTA TTC CTT ACG			
	Cytaux_cytb_Finn: ACC TAC TAA ACC TTA TTC AAG CRT T		1333 bp	Panait et al. (2021b)
	Cytaux_cytb_Rinn: AGA CTC TTA GAT GYA AAC TTC CC			
*Bartonella* spp. (16 S–23 S rRNA)	bartgd_for: GAT GAT GAT CCC AAG CCT TC		179 bp	Jensen et al. (2000)
	B1623_rev: AAC CAA CTG AGC TAC AAG CC			
*Bartonella* spp. (*gltA*)	BhCS.781p: GGG GAC CAG CTC ATG GTG G		379 bp	Norman et al. (1995)
	BhCS.1137n: AT GCA AAA AGA ACA GTA AAC A			
*Rickettsia* spp. (23 S–5S rRNA)	ITS-F: GAT AGG TCG GGT GTG GAA G		350-550 bp	Vitorino et al. (2003)
	ITS-R: TCG GGA TGG GAT CGT GTG			
Anaplasmataceae (16 S rRNA)	EHR16SD_for: GGT ACC YAC AGA AGA AGT CC		345 bp	Parola et al. (2000)
	EHR16SR_rev: TAG CAC TCA TCG TTT ACA GC			
Filarioidea (*COI*)	H14FilaCOIFw: GCC TAT TTT GAT TGG TGG TTT TGG		724 bp	Hodžić et al. (2015)
	H14FilaCOIRv: AGC AAT AAT CAT AGT AGC AGC ACT AA			

Note: Supplementary data associated with this article.

### 2.3 Phylogenetic analysis

The 18 S rRNA sequences of *C. europaeus* and the 16 S–23 S rRNA sequences of *Bartonella* spp. Were analyzed using the BLAST function on NCBI GenBank. For *C. europaeus* the *CytB* sequences were compared to those of *Cytauxzoon* spp. published by Panait et al. (2021b) to determine the species. For phylogenetic analysis, nucleotide sequences available on the NCBI GenBank database were searched by using the BLAST function, using one of the sequences obtained for each organism. The organism group was specified as Mycoplasma (taxid:2093) for the *Mycoplasma* spp. sequences and Adeleorina (taxid:75,740) for the *Hepatozoon* spp. sequences, with the number of maximum target sequences set to 5000. The sequences were aligned and sorted using the default option (FFT–NS–2) in MAFFT v.7.311 (Katoh and Standley, 2013) and sequences not covering the fragment of the sequences obtained in this study were excluded. All sequences featuring obvious sequencing errors and ambiguity characters were removed from the alignment and were excluded from the analysis. The chosen sequences included selected *Mycoplasma* spp. (based on their similarity in the alignment) and *Hepatozoon* spp. as well as other members of the suborder Adeleorina. Sequences used for analysis were uploaded to GenBank (GenBank accession numbers: ON202709-ON202711, ON180678-ON180682, OL415842-OL415874, ON380442-ON380486, ON855993-ON856037, and OL697395-OL697397).

To provide an overview of the diversity of haplotypes, Maximum Likelihood (ML) and Bayesian Inference (BI) trees were calculated for each organism based on alignments, including 158 sequences (975 nucleotide positions) for *Mycoplasma* spp. and 537 sequences (585 nucleotide positions) for *Hepatozoon* spp. Alignment gaps were removed using TrimAl v.1.3 (http://phylemon2.bioinfo.cipf.es/; Sánchez et al., 2011) and sequences were collapsed to haplotypes using DAMBE v.7.0.5.1 (Xia and Xie, 2001), leaving 84 haplotypes (969 nucleotide positions) for *Mycoplasma* spp. and 183 haplotypes (539 nucleotide positions) for *Hepatozoon* spp. As outgroup for *Mycoplasma* spp. one sequence of *Mycoplasma pneumonia* (GenBank accession number: NR041751) and for *Hepatozoon* spp. two sequences of *Adelina bambarooniae* (GenBank accession numbers: AF494058, AF494059) were used. ML bootstrap consensus trees (1000 replicates) were calculated using the W-IQ-TREE web server (http://iqtree.cibiv.univie.ac.at/; Trifinopoulos et al., 2016) applying the models TIM3+F + I + G4 for *Mycoplasma* spp. and K81u (K3P)+F + I + G4 for *Hepatozoon* spp., which were suggested as best fit for the data set in the model test according to the Bayesian inference criterion (BIC). The BI trees were calculated using MrBayes v.3.2.7 (Ronquist et al., 2012), applying the next complex model GTR + G + I, because the same models were not available in this program. The analysis was run for 10^6^ generations (Number of chains: 4), sampling every thousandth tree. The first 25% of trees were discarded as burn-in and a 50% majority-rule consensus tree was calculated based on the remaining 7500 trees.

Median-joining haplotype networks were calculated with Network 10.2.0.0 (Fluxus Technology Ltd., Suffolk, UK), applying the default settings. If only one haplotype was present, pie charts were created in Excel (2016) (Microsoft Corporation, Redmond, USA). Networks and pie charts were graphically prepared and provided with information on the countries and hosts in Network Publisher v.2.1.2.3 (Fluxus Technology Ltd., Suffolk, UK) and finalized with CorelDRAW 2021 (Corel, Ottawa, Canada). Calculation of *p*-distances was performed with MEGA version 11 (Tamura et al., 2021).

### 2.4 Statistical analysis

Binary logistic regression was conducted to test the association between detection of pathogens (summarized per genus) and tissue investigated, age and sex of the animals (each fitted as fixed categorical effects with two levels), and over time. Effects were considered statistically significant if *P* < 0.05. No multiple testing was necessary. Statistical analysis was performed using R version 4.2.0 (R Foundation for Statistical Computing, Vienna, Austria).

## 3 Results

**Fig. 3:**
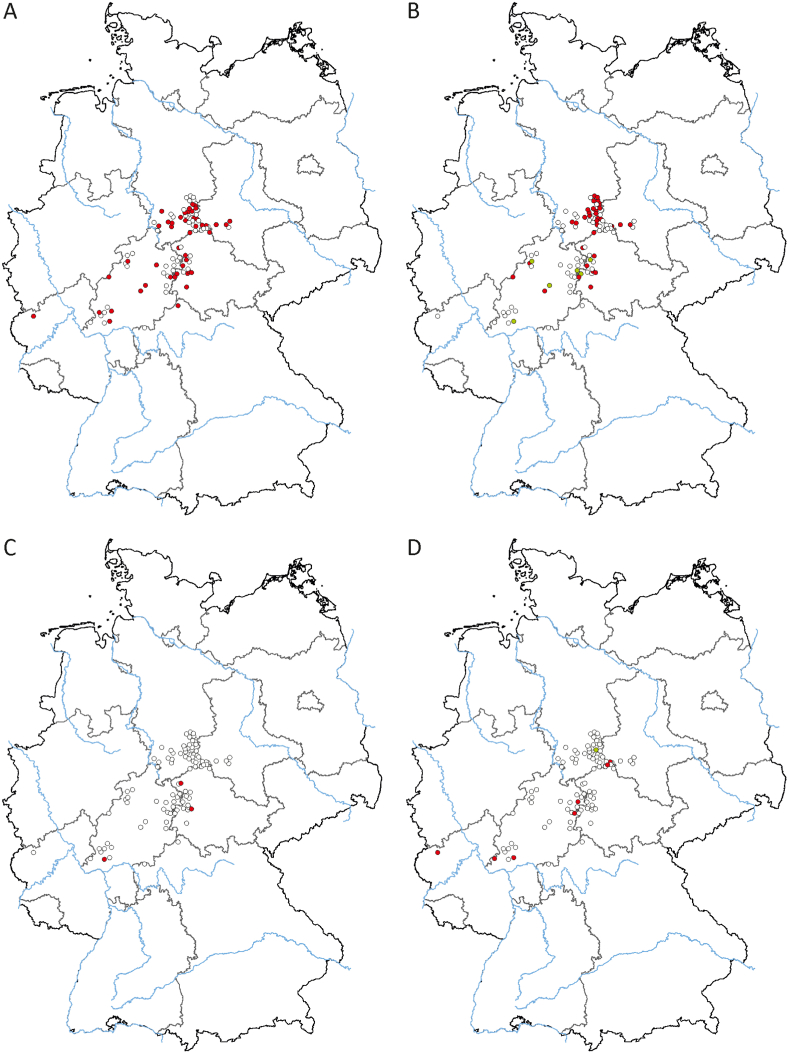
Geographical distribution of uninfected (white dots) and infected European wildcats (*Felis silvestris*) from Germany according to detected pathogens. A: red dots represent detection of *Cytauxzoon europaeus*; B: red dots represent detection of *Hepatozoon silvestris*, green dots represent detection of *Hepatozoon felis*; C: red dots represent detection of *Bartonella* spp.; D: red dots represent detection of *Candidatus* Mycoplasma haematominutum; green dots represent detection of *Mycoplasma ovis*; blue lines represent major rivers; and black lines represent borders of federal states. (For interpretation of the references to colour in this figure legend, the reader is referred to the Web version of this article.)

**Fig. 4:**
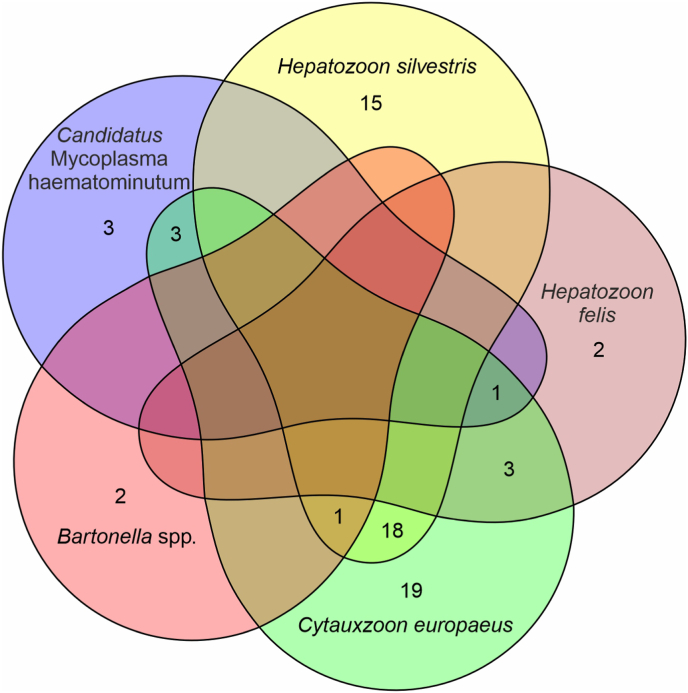
Co-infection scheme of detected pathogens, excluding *M. ovis*. Numbers represent counts of European wildcats (*Felis silvestris*) with respective pathogen(s) detected.

All individuals included in this study were European wildcats (*Felis silvestris*). The data set evaluated in this study consisted of 61 males and 35 females (comprising 25 juveniles/subadults, 70 adults, and one of unknown age). Pathogens detected were *Candidatus* Mycoplasma haematominutum (n = 7; 7.29%), *Mycoplasma ovis* (n = 1; 1.04%), *Hepatozoon* s*ilvestris* (n = 34; 35.42%), *H. felis* (n = 6; 6.25%), *Cytauxzoon europaeus* (n = 45, 46.88%) and *Bartonella* spp. (n = 3; 3.13%). All PCRs for Filarioidea, Anaplasmataceae, and Rickettsiales were negative (Fig. 3). In total, pathogens were found in 67/96 (69.79%) wildcats. One pathogen only was documented in 40/96 (41.97%) cats, two different pathogens were found in 25/96 (26.04%), and three different pathogens were detected in 2/96 (2.08%) animals (Fig. 4). Logistic regression did not detect any association between pathogen occurrence and tissue investigated, and age and sex of the animals. There was a statistically significant increase in detecting *C. europaeus* (*P* = 0.038, McFadden R^2^ = 0.05) over time, but no other association over time was detected.

**Fig. 5:**
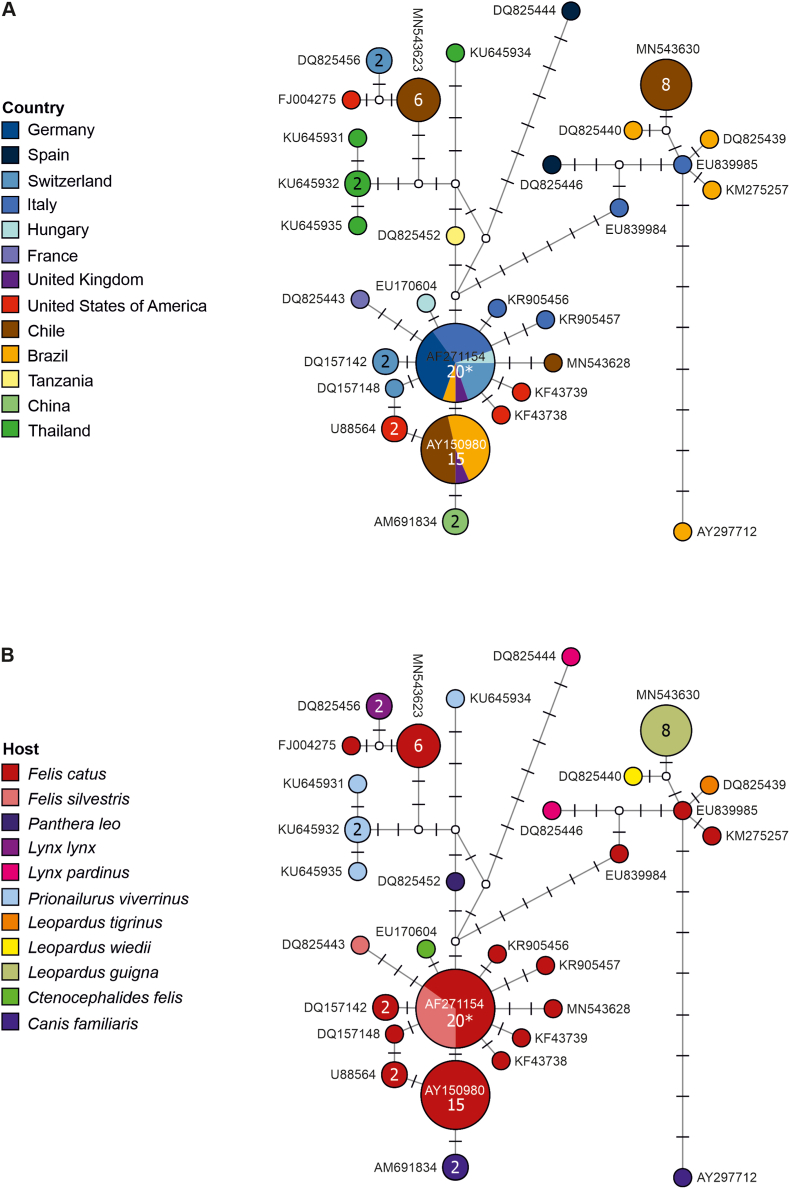
Median Joining haplotype network of the 16 S rRNA sequences (983 nucleotide positions) of *Candidatus* Mycoplasma haematominutum showing the geographical distribution (A) and the reported hosts (B). Circles represent haplotypes; numbers within the circles represent the number of individuals, if no number is shown, then only one individual is represented; labels next to circles specify representative GenBank accession numbers of the haplotypes, white circles represent intermediate nodes; bars on branches interconnecting haplotypes represent the number of substitutions; and asterisks mark haplotypes containing the individuals obtained in the present study.

The genetic analysis of *Candidatus* Mycoplasma haematominutum revealed one haplotype identical to a haplotype found in domestic cats in Switzerland, Italy, Hungary, the United Kingdom, and Brazil (Fig. 5). This haplotype was placed within the clade (BI posterior probability (BI pp): 1; ML bootstrap value (ML bs): 100) of other *Candidatus* Mycoplasma haematominutum sequences in the consensus tree (Suppl. 1). The sequence of *M. ovis* obtained in this study showed 100% identity to a *M. ovis* sequence found in a goat (*Capra hircus*) from China (GenBank accession number: KU983745).

**Fig. 6:**
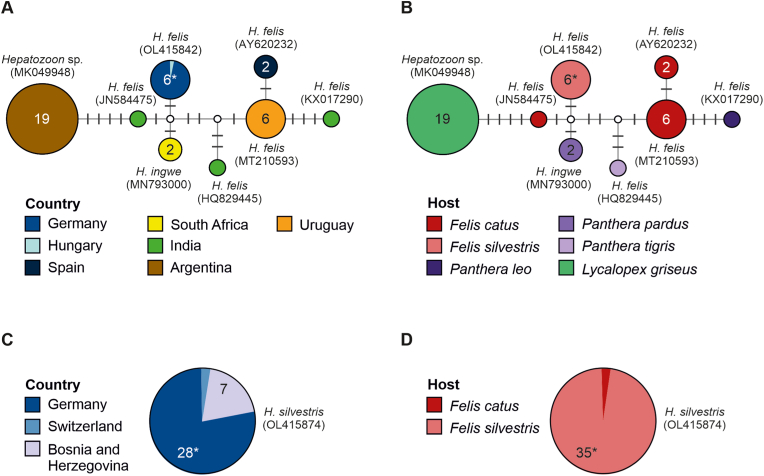
Median Joining haplotype network of the 18 S rRNA sequences (561 nucleotide positions) of *Hepatozoon felis* (A, B) and pie chart of the 18 S rRNA gene (572 nucleotide positions) of *Hepatozoon silvestris* (C, D) showing the geographical distribution (A, C) and the reported hosts (B, D). Circles represent haplotypes; numbers within the circles represent the number of individuals, if no number is shown, then only one individual is represented; labels next to circles specify organism name and representative GenBank accession numbers of the haplotypes, white circles represent intermediate nodes; bars on branches interconnecting haplotypes represent the number of substitutions; and asterisks mark haplotypes containing the individuals obtained in the present study.

The sequences of *H. silvestris* were 100% identical to the haplotype detected in wildcats in Bosnia and Herzegovina and one domestic cat in Switzerland. The sequences of *H. felis* were 100% identical to the haplotype found in a wildcat from Hungary (Fig. 6). In the consensus tree, *H. felis* and *H. silvestris* were placed in a clade (BI pp: 0.65; ML bs: 75) together with *Hepatozoon* spp. mainly found in Canidae, Suidae, and Mustelidae (Suppl. 2). Within that clade, the two species were not closely related, and most sequences of *H. felis* were placed in a separate clade in both the BI tree (BI pp: 0.65) and the consensus tree (BI pp: 0.93; ML bs: 99), except for two sequences that fell outside this clade. Two *H. felis* sequences were placed in another distinct clade (BI pp: 0.92; ML bs: 99) together with *H. luiperdjie*.

The *18S* rRNA sequences of *C. europaeus* showed high similarity to other European *Cytauxzoon* spp. (99.77–100% identity to *C. europaeus* with the GenBank accession number MT904044). The alignment of *CytB* sequences with *C. europaeus* (GenBank accession number: MT916191), *C. otrantorum* (GenBank accession number: MT916204), *C. banethi* (GenBank accession number: MT916193), and *C. felis* (GenBank accession number: MT916203) showed the highest similarity to *C. europaeus* with a *p*-distance < 0.011.

The three *Bartonella* spp. sequences obtained in this study were distinct from each other and showed 100% identity to *Bartonella* sp. found in a yellow-necked mouse (*Apodemus flavicollis*) from Slovakia (GenBank accession number: KX267683), 100% identity to *Bartonella* sp. found in a common mole (*Microtus arvalis*) from Poland (GenBank accession number: GU338968) and 100% identity to *Bartonella* sp. found in a bank vole (*Clethrionomys glareolus*) from Slovakia (GenBank accession number: KX267679), respectively.

## 4 Discussion

The large sample size of the present study allowed us to obtain an overview of blood-borne pathogens harboured by wildcats in Germany. The pathogens detected and their prevalence are comparable to those obtained by studies on wildcats from other parts of Europe using molecular genetic tools (Willi et al., 2007, 2022; Gallusová et al., 2016; Veronesi et al., 2016; Hodžić et al., 2018a; Panait et al., 2021b).

Our results of *Hepatozoon* spp. are comparable with the results in wildcats from Bosnia and Herzegovina, where *H. felis* (3/9), *H. silvestris* (2/9), and unidentified *Hepatozoon* species (2/9) were detected (Hodžić et al., 2018a). In the same study, *Cytauxzoon* sp. (10/18) was found, which is consistent with results in wildcats from Romania (9/12) and with our study (Gallusová et al., 2016; Hodžić et al., 2018a). *Cytauxzoon* sp. Was also shown to be present in wildcats in Italy (4/21), albeit with a lower prevalence (Veronesi et al., 2016). Panait et al. (2021b) analyzed European *Cytauxzoon* spp. in more detail and described three new species. They found *C. europaeus* in wildcats from Germany (30/46), Romania (9/31), the Czech Republic (5/11), and Luxembourg (9/13). This is also comparable with the findings of *C. europaeus* in wildcats from France (10/34) and with our results (Willi et al., 2022).

Rickettsiales such as *Anaplasma phagocytophilum* are widespread tick-borne pathogens in many mammals in Europe, and have also been reported in domestic cats from Germany. However, we did not detect these pathogens in our study (Stuen, 2007; Morgenthal et al., 2012; Bergmann et al., 2015; Bergmann and Hartmann, 2017; Schäfer et al., 2022). Likewise, García-Pérez et al. (2016) did not detect Anaplasmataceae in wildcats from Spain (0/8), although the sample size may have been too small for detection. This is not the case with the sample size in our study. Considering that serological detection revealed a higher prevalence than molecular detection in studies conducted in domestic cats, our results may suggest few active infections with this pathogen, rather than an absence of infection in wildcats (Morgenthal et al., 2012; Schäfer et al., 2022).

*Candidatus* Mycoplasma haematominutum was detected in our study as well as in wildcats from France (6/13), where *Candidatus* Mycoplasma turicensis (11/31) was also found (Willi et al., 2007). In the study by Hodžić et al. (2018a) mentioned above *Mycoplasma* spp., which were genetically distinct from *Candidatus* Mycoplasma haematominutum, were found in wildcats from Bosnia and Herzegovina (4/18). Surprisingly, we detected *M. ovis* in a wildcat in our study. Since *M. haemofelis* was used as a positive control, contamination of the sample is unlikely. This pathogen is usually found in sheep and other small ruminants, but it was also recently reported in horses from Iran (Kalantari et al., 2020). *M. ovis* was likely only transiently present in the blood of the one wildcat from our study, as there are no other reports of *M. ovis* in carnivores to the authors’ knowledge.

*Bartonella* spp. sequences obtained in our study were distinct from each other but were all 100% identical to sequences found in rodents. It is also possible that these pathogens were temporarily present in the blood, or that transmission to wildcats occurred through predation. This hypothesis, however, would need further investigation. Generally, fleas are known to play an important role in the spread of *Bartonella* spp. and are also discussed as vectors for haemotrophic *Mycoplasma* spp. (Millán et al., 2021). In fact, *Candidatus* Mycoplasma haematominutum was detected in cat fleas (*Ctenocephalides felis*) in Hungary (Hornok et al., 2010). Findings of Millán et al. (2021) support this theory, as co-infection of *Bartonella* spp. and haemotrophic *Mycoplasma* spp. often occur, although ingestion of the cat flea does not seem to play a role in transmission (Woods et al., 2006). This co-infection was not detected in our study, possibly due to the low prevalence of *Bartonella* spp. In the same review, Millán et al. (2021) state that detection of haemotrophic *Mycoplasma* spp. is also dependent on the tissue investigated, being more prevalent in blood compared to spleen tissue, probably because the pathogen is eliminated more quickly from the spleen compared to the blood. Likewise, in our study, *Mycoplasma* spp. Was detected more often in the blood compared to spleen tissue, although this difference was not statistically significant. Modelling of transmission pathways for *Candidatus* Mycoplasma haematominutum suggests a concurrent role of vectors as well as direct transmission (Kellner et al., 2018).

Similarly, there are known routes of transmission for Piroplasmida, for example by hard ticks, but they are not fully elucidated for all species (Giannelli et al., 2017b; Thomas et al., 2018). Based on the overlapping distribution of the pathogens detected in our study, *Ixodes ricinus*, *Dermacentor reticulatus*, *Ixodes hexagonus*, and *Ixodes inopinatus* could act as possible vectors (Rubel et al., 2021), but other routes of transmission are also suggested to play a role, such as direct transmission through bites or diaplacentar transmission (Hornok et al., 2013; Hodžić et al., 2018a, 2018b). The high number co-infections with *C. europaus* and *H. silvestris* might indicate a common transmission route for these pathogens, but since these pathogens were the most prevalent, a high rate of co-infection is expected.

Although climate change is considered to promote the distribution of vectors (Harrus and Baneth, 2005; Aguirre, 2009; Cunze et al., 2022), for most pathogens we found no evidence of a trend over time for possible or definite vector transmission. However, it could be that a trend in this and other transmission routes, was not yet detectable, but will become apparent as climate change progresses. In that case, this study will provide essential baseline data. Nevertheless, an increase over time was observed in *C. europaeus*, although unknown confounding factors may not have been accounted for in the model, as indicated by the low McFadden R^2^. Although Willi et al. (2022) demonstrated that *C. europaeus* could be detected in wildcat samples between 1995 and 1996 and for this reason do not consider this pathogen as emerging, an increase in prevalence in wildcats over time may have led to spill over into domestic cats and therefore explain the recent more frequent detection in domestic cats Legroux et al. (2017); Panait et al. (2021b); Willi et al. (2022).

Interestingly, *H. silvestris* was more widespread in central Germany than *H. felis*, which was detected more in the western part of Germany. This distribution might reflect the separation of the German wildcat population into a western and central population (Mattucci et al., 2016).

The phylogenetic analysis of *Mycoplasma* spp. and *Hepatozoon* spp. sequences focused on generating a network to illustrate the distribution of haplotypes according to hosts and countries with closely related sequences. For this reason, *M. haemofelis* and *Candidatus* Mycoplasma turicense were not included in our phylogenetic tree, due to their dissimilarity, although these pathogens have been found in wild felids and their phylogenetic relation was described by Willi et al. (2007). For *Hepatozoon* spp., the BI tree only supported the clade of *H. felis* with a posterior probability of 0.65, and the clade was not supported in the ML tree. However due to the high similarity of the sequences this clade was chosen for the network analysis.

The *H. felis* haplotype found in the present study and in a wildcat from Hungary was not closely related to the only other haplotype found in Europe in domestic cats from Spain (Criado-Fornelio et al., 2006; Hornok et al., 2022). Furthermore, Hornok et al. (2022) described two distinct genotypes of *H. felis* in wildcats from Hungary. Comparison with the consensus tree calculated in the present study shows that these genotypes refer to the clade containing the *H. felis* sequences obtained here, and to the clade containing sequences of *H. luiperdjie* described by van As et al. (2020) in a leopard (*Panthera pardus*). These findings support the hypothesis that *H. felis* is not a phylogenetically well-defined species, but rather a species complex that requires further investigation (Hodžić et al., 2017; van As et al., 2020; Hornok et al., 2022).

Apart from one report in wildcats from France (Willi et al., 2007), which had a different haplotype, this is the first report of this *Candidatus* Mycoplasma haematominutum lineage in wildcats. All sequences from the present study belong to the same haplotype, which is also the major haplotype reported, but has only been detected in domestic cats until now (Tasker et al., 2001; Willi et al., 2006; Hornok et al., 2008; Aquino et al., 2014). The haplotype of *H. silvestris* reported in the present study is identical to the only haplotype reported so far in wildcats from Bosnia and Herzegovina, and also in a domestic cat from Switzerland (Hodžić et al., 2017; Kegler et al., 2018). This indicates that wildcats and domestic cats do share blood-associated pathogens.

The clinical impact of the pathogens detected in wildcats is unknown. Although there are case reports in domestic cats with severe disease (Hornok et al., 2008; Legroux et al., 2017; Kegler et al., 2018; Basso et al., 2019), the high prevalence of *Candidatus* Mycoplasma haematominutum, *Hepatozoon* spp., and *C. europaeus* more likely indicate asymptomatic infection in wildcats. This theory is supported by other studies reporting high prevalence without clinical disease (Willi et al., 2006; Grillini et al., 2021).

Hodžić et al. (2018a), as well as a study performed in Romania (Panait et al., 2021a), described the presence of *Babesia* spp. in wildcats, yet we failed to detect this parasite in the present study. In our study, Piroplasmida and other Apicomplexa were detected by a nested PCR, and detection of *Hepatozoon* spp. or *Cytauxzoon* spp. might have interfered with the detection of *Babesia* spp. This interpretation is contradicted by the fact that in the study by Hodžić et al. (2018a) the same method was used, and *Babesia* sp. Was detected in a sample that was also positive for *Cytauxzoon* sp. Another explanation might be that *Babesia* spp. associated with felids are not yet widespread in Europe (Penzhorn and Oosthuizen, 2020). Similarly, Filarioidea, such as *Dirofilaria* spp. Were not detected in our study, which is most likely due to the fact that though *Dirofilaria* spp. has been described in mosquitoes and vertebrate hosts in Germany, the prevalence is not considered to be high (Fuehrer et al., 2021). Romania is a highly endemic country for *Dirofilaria* spp., and *D. immitis* has been detected in a wildcat there (Ionică et al., 2017).

In conclusion, this study provides information on the prevalence of blood-associated pathogens in wildcats from Germany. Considering that the wildcat is an endangered species, the data can be of importance for wildlife conservation. The results are also valuable for veterinarians, as free-ranging domestic cats roam in the same area as wildcats and are therefore at risk of infection. However, additional studies are needed to elucidate the route of transmission and the clinical impact of these pathogens.

## Data availability statement

The data presented in this study are contained within the article and supplementary material (Supplementary file).

## Funding

KH was supported by the project Nr. CZ.02.1.01/0.0/0.0/16_019/0000787 ‘Fighting Infectious Diseases' provided by the 10.13039/501100001823Ministry of Education, Youth and Sports of the Czech Republic. All other researchers received no specific grant from funding agencies in the public, commercial, or not-for-profit sectors.

## Declaration of competing interest

None.
