# Supplementary material for: Molecular analysis of blood-associated pathogens in European wildcats (Felis silvestris silvestris) from Germany
Source: Int J Parasitol Parasites Wildl. 2022 Sep 3;19:128–37. doi: 10.1016/j.ijppaw.2022.08.012 (PMC9477852; doi:10.1016/j.ijppaw.2022.08.012)
Supplement: Multimedia component 1 [file mmc1.pdf]

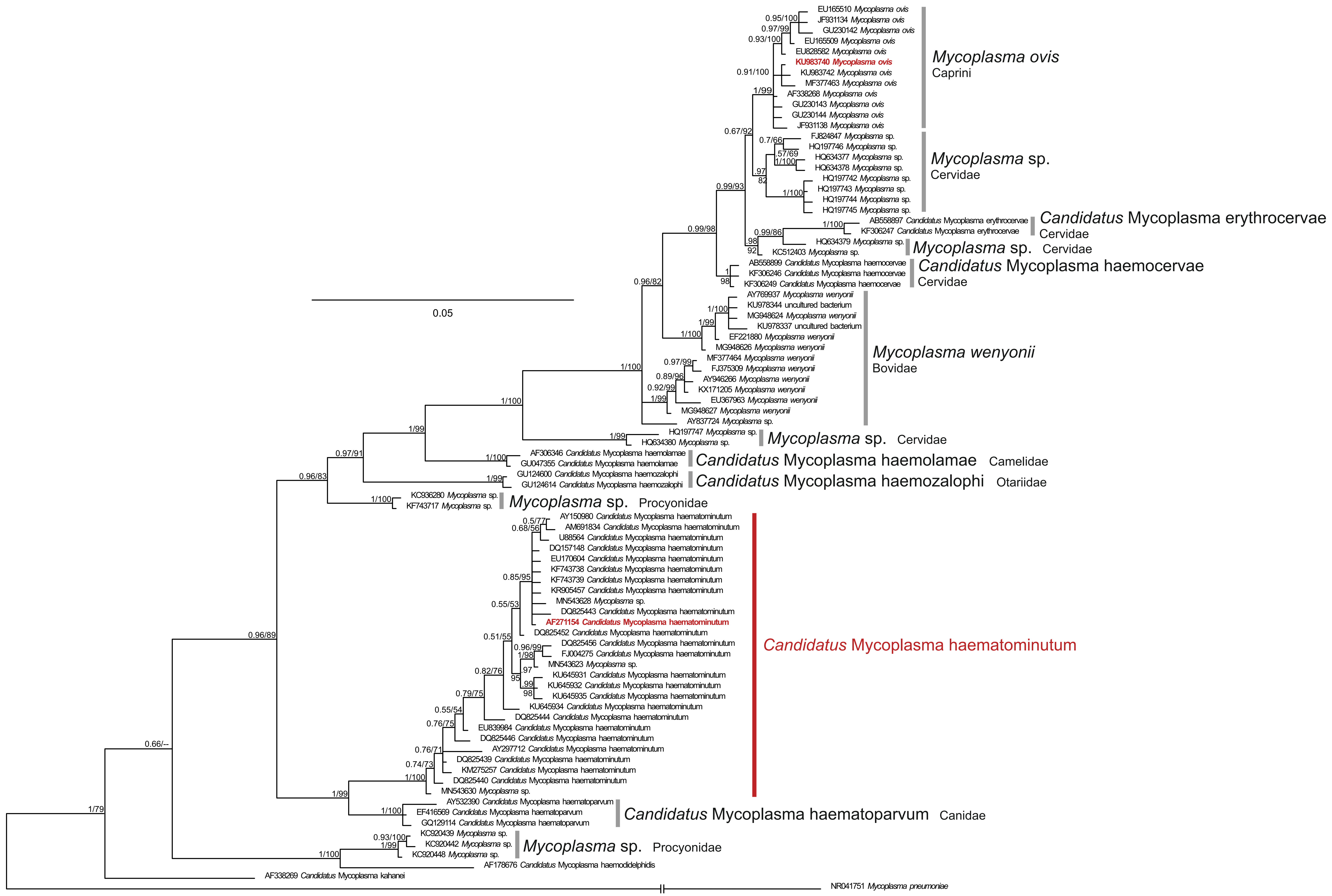

Supplement 1. Bayesian interference (BI) tree featuring *16S* rRNA sequences (969 nucleotide positions) of selected *Mycoplasma* spp. Nodes are marked with BI posterior probabilities and ML bootstrap values. Clades which are marked in red were used for calculation of the median-joining haplotype networks containing the sequences obtained in this study. Scale bar indicates the expected mean number of substitutions per site according to the model of sequence evolution applied.
