# Supplementary figures and images for: Molecular analysis of blood-associated pathogens in European wildcats (Felis silvestris silvestris) from Germany
Source: Int J Parasitol Parasites Wildl. 2022 Sep 3;19:128–37. doi: 10.1016/j.ijppaw.2022.08.012 (PMC9477852; doi:10.1016/j.ijppaw.2022.08.012)

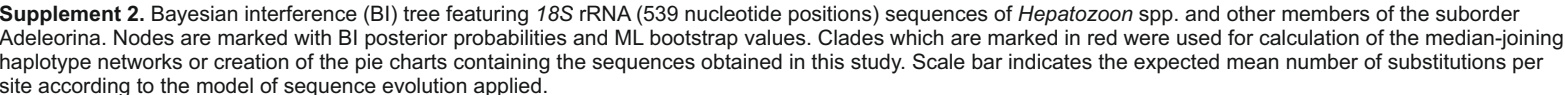

Supplement: Multimedia component 2 [file mmc2.pdf]
